# Retrospective analysis of changes in the anterior corneal surface after Q value guided LASIK and LASEK in high myopic astigmatism for 3 years

**DOI:** 10.1186/1471-2415-12-15

**Published:** 2012-06-18

**Authors:** Hui Huang, Jianguo Yang, Huijing Bao, Shaorong Chen, Beibei Xia, Jun Zou

**Affiliations:** 1 Department of Ophthalmology, Shanghai Sixth People's Hospital, Shanghai Jiaotong University, 600 Yishan Road, Shanghai, 200233, People’s Republic of China

**Keywords:** Corneal aberrations, Q-value guided, LASIK, LASEK, Asphericity

## Abstract

**Background:**

To compare the corneal high-order aberrations (HOAs), asphericity and regularity after Q-value guided laser *in situ* keratomileusis (LASIK) and laser epithelial keratomileusis (LASEK) in high myopic astigmatism.

**Methods:**

In this retrospectively comparative study, we measured the corneal HOAs, asphericity indices (Q values) and corneal regularity indices preoperatively and 36 months postoperatively in 70 eyes (35 patients) with Q-value guided surgeries. All the patients with high myopic astigmatism were divided into two groups which included 34 eyes underwent LASIK and 36 eyes underwent LASEK procedures. The main impact factors of the high-order aberrations were also analyzed.

**Results:**

In the two groups, the efficacy index was more than 1.00 and safety index approached 1.00 at year 3 postoperatively. Statistically significant (P < 0.05) increased in Q values and main corneal HOAs (spherical aberrations and coma) following Q-value guided LASIK and LASEK procedures. Spherical aberrations increased more in the LASEK group and there was statistically difference compared to the LASIK group (P < 0.05). LASEK had better effects in correcting corneal astigmatism (P < 0.05). All the corneal regularity indices after surgeries increased and there was no significant difference (P = 0.707, P = 0.8 and P = 0.224, respectively) between the two groups. The main impact factors of spherical aberration included the optic zone size, changes of Q value, surgical procedure and the corrected refraction.

**Conclusions:**

In high myopic astigmatism, Q-value guided ablation showed good safety, efficacy and predictability. Q value, regularity indices, spherical aberration and coma increased in both LASIK and LASEK procedures. Astigmatism could be corrected more effectively by LASEK but greater spherical aberration could be created. The difference might be related to the different healing mechanisms. Optic zone size and the corrected refraction might be the main influence factors on the anterior corneal high order aberrations.

## Background

Laser *in situ* keratomileusis (LASIK) was reported clinically in 1990 [1] and was still a commonly performed surgery all over the world. Laser epithelial keratomileusis (LASEK), as a surface ablation technique, has gained popularity in recent years. However, many previously published papers have indicated that there was occasional poor postoperative quality of vision after conventional refractive surgery such as glare and halos [2]. In recent years, customized ablation surgery emerged to improve visual quality. Q value guided ablation, as one of the customized surgical procedures, has been reported clinically in recent years. It would be interesting to know its efficacy and safety in correcting high myopic astigmatism and how it changed the shape of the cornea. The main goals of this study were to compare visual acuity, corneal high-order aberrations and changes in the anterior corneal surface between Q-value guided LASIK and LASEK.

## Methods

We reviewed the records of patients who underwent Q value guided refractive surgery (including LASIK and LASEK) at Shanghai Sixth People's Hospital during 2007 and who had a postoperative follow-up at year 3 after surgeries. Aspheric LASIK was performed on 34 eyes of 17 patients (8 eyes of 4 males, 26 eyes of 13 females), and aspheric LASEK was performed on 36 eyes of 18 patients (4 eyes of 2 males, 32 eyes of 16 females). The study protocol was approved by Ethics Committees of the hospitals and shanghai Jiaotong University. Written informed consent was obtained from each subject. Patient demographics are summarized (Table 1). All the patients in this study were given information about the surgical procedure and possible complications. Inclusion criteria were the patients without pathologic myopia or other eye disease and without related systemic diseases, such as diabetes mellitus, preoperative manifest refraction greater than -6.00 diopters (D) and astigmatism up to -2.50 (D). Exclusion criteria included patients whose uncorrected distance visual acuity (UDVA) was less than 20/20 (6/6) with suboptimal visual outcome, complications following surgery such as dry eye or haze. All surgeries were performed by one surgeon (J.Z), who had extensive experience with refractive surgery.

**Table 1:** Patient demographics

	**LASIK**	**LASEK**	**P**
**Age(y)**	34.41 ± 5.29	32.72 ± 5.10	0.18
**MRSE(D)**	-7.32 ± 1.41	-6.94 ± 1.50	0.27
**CDVA(logMAR)**	-0.06 ± 0.03	-0.06 ± 0.06	0.78
**Q30**	-0.35 ± 0.15	-0.35 ± 0.15	0.70
**Optic zone(mm)**	6.71 ± 0.24	6.63 ± 0.48	0.38

*MRSE*(manifest refraction spherical equivalent), *CDVA* (corrected distance visual acuity).

All patients underwent refractive surgeries using the ALLEGRETTO WAVE Eye-Q 400-Hz excimer laser ((Wave-Light AG, Erlangen, Germany) with the fine adjusted-customized ablation treatment (F-CAT) algorithm. The targeted Q value was set according to the mean of Q_1_ and Q_2_, which were calculated by the corneal eccentricity of two main meridians within 30 degrees. The preoperative K-value and Q-value determined by corneal topography were used for aspheric ablation.

Surgical procedure: In the LASIK procedure, a corneal flap (thickness of approximately 110-130 μm) was created using an auto mechanical microkeratome (Moria 90, France). In the LASEK procedure, the corneal epithelium was incised with a trephine placed centrally, and 20% alcohol was applied for 15-20 s and then detached an epithelial flap. After these procedures, laser ablation was performed to manifest the refraction.

All patients were examined before and at year 3 after the surgery. The evaluations included Q value changes of the anterior corneal surface, safety and efficacy of the operation, residual refractive errors, topography regularity indices (including index of surface variance [ISV], index of vertical asymmetry [IVA], index of height asymmetry [IHA]) and corneal higher order aberrations (spherical aberration and coma). Safety was evaluated in terms of a calculated safety index (= mean postoperative corrected visual acuity/mean preoperative corrected visual acuity). Efficacy was determined by calculating an efficacy index (= mean postoperative uncorrected visual acuity/mean preoperative uncorrected visual acuity).

Corneal topography was recorded using an Allegro topolyzer (Allegro topolyzer, Wavelight, Germany). The topographic maps of each eye were examined by one observer and three topographic maps were recorded for each eye. From the corneal topography, the wavefront errors of the anterior corneal surface at 6 mm pupils were calculated and decomposed into Zernike polynomials to the 7th order. Z_n_^m^ is the Zernike coefficient of radial order n and angular frequency m. Spherical aberration was expressed as Z_4_^0^ and coma was expressed as Z_3_^1^.

### Statistical analysis

All parameters were recorded as mean ± standard deviation. Student *t*-test was performed to determine statistically significant differences. The Pearson correlation and multiple linear regression was calculated to determine relevant factors analysis in stepwise method. A P value <0.05 was considered statistically significant. Statistics were calculated using SPSS Version 17.0 (IBM).

## Results

### Visual acuity

Visual acuity and refraction conditions postoperatively are noted in Table 2. The mean safety index after aspheric LASIK and aspheric LASEK were 1.03 ± 0.14 and 1.03 ± 0.11, respectively, with no statistically significant difference between the groups (P = 0.83). The mean efficacy index after aspheric LASIK and aspheric LASEK were 0.98 ± 0.14 and 1.00 ± 0.15 respectively, with no statistically significant changes between two groups (P = 0.59).

**Table 2:** Visual acuity and refraction postoperatively

	**Total**	**LASIK**	**LASEK**	**P**
**UDVA(logMAR)**	-0.05 ± 0.06	-0.05 ± 0.06	-0.06 ± 0.06	0.39
**Refraction errors(D)**	+0.68 ± 0.37	+0.62 ± 0.38	+0.74 ± 0.36	0.16
**safety indices**	1.03 ± 0.13	1.03 ± 0.14	1.03 ± 0.11	0.83
**efficacy indices**	0.99 ± 0.15	0.98 ± 0.14	1.00 ± 0.15	0.59

### Refraction errors (D)

**Figure 1:**
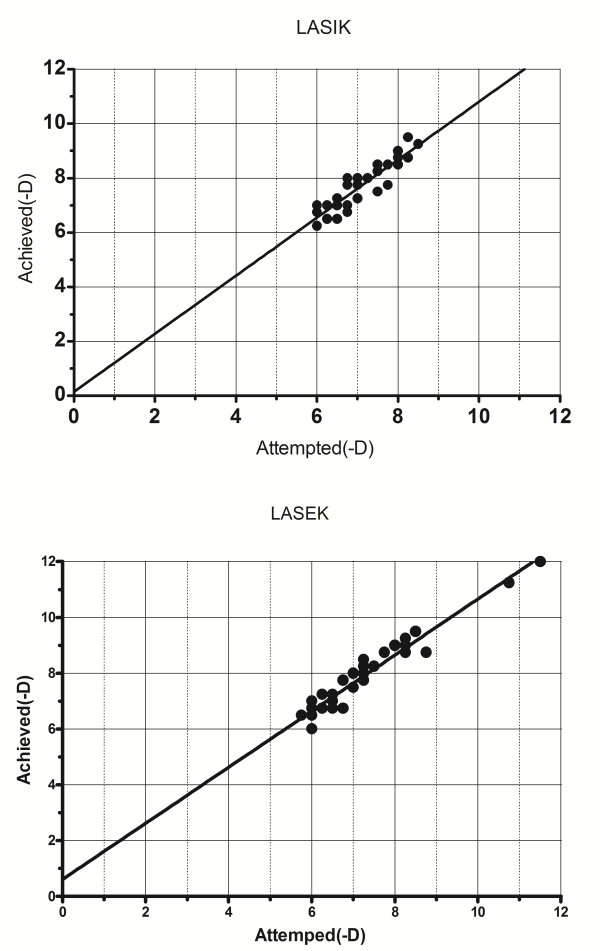
**The mean refraction errors after aspheric LASIK and LASEK.** Attempted spherical equivalent refraction versus achieved manifest refraction spherical equivalent (MRSE) in the Q-value guided LASIK group and LASEK group 3 years after surgery. LASIK: y = 1.07x + 0.15, R^2^ = 0.81; LASEK: y = 1.00x + 0.6, R^2^ = 0.91.

The mean refraction errors after aspheric LASIK and aspheric LASEK were +0.62 ± 0.38 (D) and +0.74 ± 0.36 (D), with no statistically significant difference (P = 0.16) (Figure 1).

### Q value changes

**Figure 2:**
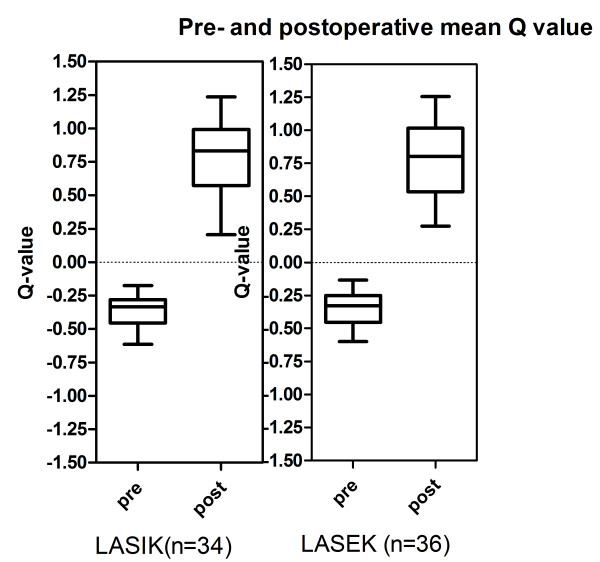
**The mean Q value for the two groups.** The mean Q value increased from -0.35 ± 0.15 to 0.79 ± 0.33 in LASIK and from -0.35 ± 0.15 to 0.79 ± 0.30 in LASEK group.

The mean Q value for the LASIK group and the LASEK group were noted in Figure 2. There were no differences in Q values postoperatively between the two groups (P = 0.838 and P = 0.759, respectively).

### Topographic maps analysis

**Figure 3:**
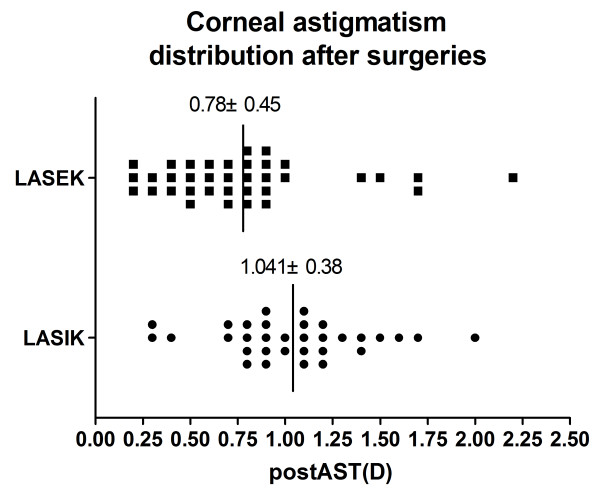
**The mean AST at year 3 after surgery.** The postoperative mean AST was 1.041 ± 0.38 in the LASIK group and 0.78 ± 0.45 in the LASEK group, with a statistically significant difference (P < 0.005). But there were no significant differences in changes of AST between the two groups.

The AST index was calculated as AST = simK_2_-simK_1_. It indicates astigmatism from the anterior surface of the cornea. Before surgery, the mean AST was 1.29 ± 0.56 in the LASIK group and 1.15 ± 0.71 in the LASEK group (P = 0.992). At year 3 after surgery, the mean AST was noted in Figure 3.

Topographic maps showed various indices calculated with topographic map data that could reflect the regularity of the corneal anterior surface, including ISV, IVA and IHA. These indices were achieved from the Allegro topolyzer using the indices mode. In this study, statistically significant (P < 0.001) changes in all indices following surgery (ISV, IVA, IHA) were noted. There were no statistically significant differences (P > 0.05) between the two Q value guided operations (Table 3).

**Table 3:** Corneal regularity indices

	**Pre**	**Post**	**Changes**	**P**
**ISV**				
**LASIK**	23.06 ± 4.76	54.25 ± 9.15	34.25 ± 18.15	<0.001
**LASEK**	23.72 ± 6.31	53.08 ± 15.17	29.36 ± 16.05	<0.001
**P**	0.622	0.707	0.709	
**IVA**				
**LASIK**	0.15 ± 0.61	0.41 ± 0.14	0.29 ± 0.19	<0.001
**LASEK**	0.15 ± 0.07	0.40 ± 0.16	0.26 ± 0.15	<0.001
**P**	0.945	0.800	0.938	
**IHA**				
**LASIK**	5.65 ± 4.32	23.51 ± 12.69	19.76 ± 16.33	<0.001
**LASEK**	7.17 ± 5.39	27.47 ± 13.75	20.29 ± 13.79	<0.001
**P**	0.199	0.224	0.454	

*P < 0.05, statistically significant.

### Topography-derived wavefront errors

**Figure 4:**
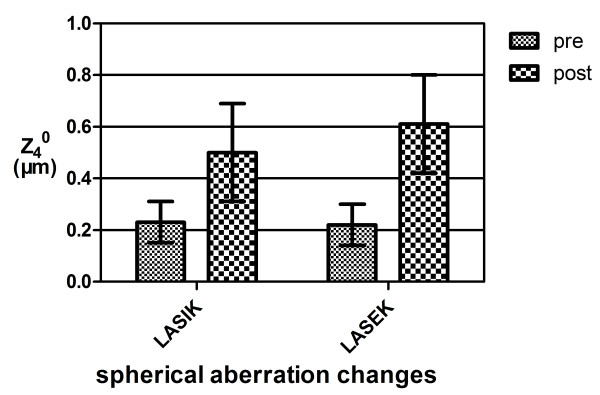
**The mean spherical aberration between the two groups.** Before surgery, the mean spherical aberration (Z_4_^0^) was 0.23 ± 0.08 μm in the LASIK group and 0.22 ± 0.08 μm in the LASEK group (P = 0.129), without difference. Up to 3 years after surgery, the mean spherical aberration was 0.50 ± 0.19 μm in LASIK and 0.61 ± 0.19 μm in LASEK, differed significantly between the two groups (P = 0.029).

Before surgery, the mean spherical aberration (Z_4_^0^) was 0.23 ± 0.08 μm in the LASIK group and 0.22 ± 0.08 μm in the LASEK group (P = 0.129). Up to 3 years after surgery for both procedures, the mean spherical aberration differed significantly between the two groups (P = 0.029). (Figure 4) The mean spherical aberration increased in both groups but was greater in the Q value guided LASEK group compared to the LASIK group. The mean coma increased in both groups and there were no differences between aspheric LASIK and aspheric LASEK procedures (P = 0.315).

### Relevant factors analysis

Pearson correlation coefficients of postoperative spherical aberration(Z40) and coma(Z31) were noted in Table 4 and Table 5 respectively. According to correlation coefficients, we analyzed impact factors of spherical aberration and coma using stepwise multiple regression analysis. The multivariate linear model of spherical aberration (Z_4_^0^) and coma (Z_3_^1^) were noted in Table 6 and Table 7 respectively.

**Table 4:** Spherical aberration correlation coefficient

	**correlation coefficient**	**P**
**Sex**	0.241	0.048
**Group**	0.265	0.029
**Optic zone**	-0.672	0.000
**Pre MRSE**	-0.296	0.014
**Pre AST**	-0.272	0.025
**Change Q**_**30**_	0.242	0.047
**Change Diopter**	0.317	0.008

**Table 5:** Coma correlation coefficient

	**correlation coefficient**	**P**
**Sex**	0.374	0.002
**Optic zone**	-0.315	0.009
**Pre MRSE**	-0.367	0.002
**Change Diopter**	0.363	0.002
**Change Q**_**30**_	0.248	0.041

**Table 6:** Spherical aberration regression coefficient

	**Unstandardized coefficient**	**Std. Error**	**Standardized coefficient**	**t**	**P**
**Constant**	2.187	0.312		7.008	0.000
**Optic zone**	-0.341	0.041	-0.655	-8.292	0.000
**Change Q**_**30**_	0.241	0.069	0.279	3.500	0.001
**Group**	0.083	0.031	0.207	2.647	0.00
**Change Diopter**	0.032	0.013	0.192	2.408	0.019

R^2^ = 0.623.

**Table 7:** Coma regression coefficient

	**Unstandardized coefficient**	**Std. Error**	**Standardized coefficient**	**t**	**P**
**Constant**	0.740	0.624		1.186	0.024
**Sex**	0.269	0.080	0.350	3.374	0.001
**Pre MRSE**	-0.082	0.025	-0.337	-3.237	0.002
**Optic zone**	-0.165	0.080	-0.218	-2.073	0.042

R^2^ = 0.323.

## Discussion

The Q value, which reflects corneal asphericity, is negative for most eyes and not related to the degree of myopia [3]. However, conventional surgery could make the cornea undergo a pathological topographical change, from its initially prolate shape (Q < 0) with a steeper central area and flat peripheral area to an oblate shape (Q > 0) with a flat center and steep periphery [4-6]. Q value guided surgery aimed to minimize changes of the corneal anterior surface asphericity in order to reduce the spherical aberration, which impacts mostly on visual quality.

Most of previous papers about Q value guided ablation were focused on short or medium-term study, correcting low myopia or medium myopia, or comparing aspheric and conventional spheric ablation. Then what about correcting high myopic astigmatism? How the parameters of the anterior corneal surface changed following Q value guided LASIK and LASEK?In the present study, Q value guided LASIK and LASEK showed good efficacy, safety, and predictability in correcting high myopic astigmatism. There were no statistically significant differences between the two groups in the residual refractive errors. Q value guided ablations demonstrated a high safety profile, with no eyes losing lines of CDVA.

After 3 years follow-up, we found the Q value of the anterior surface was inevitably shifted from negative to positive. Consequently, cornea became oblate which means it was relatively flatter in central cornea. The reason might be the differences between ablation algorithm and actual ablation or the setting of Q value target was still not suitable in the present study. For the Q value guided ablation, there were still some controversies over what the ideal target Q should be [7]. Aspheric ablation patterns, such as wavefront optimized algorithm in our excimer laser platform. The target Q was set -0.20 in wavefront optimized algorithm, without the need for customized treatment in every patient [8]. In previous studies, the wavefront-optimized algorithm of the Allegretto Wave Eye-Q 400-Hz excimer laser platform showed good visual and refractive results [9,10]. Anera et al. [11] Compared wavefront optimized (standard) and Q-optimized (F-CAT) algorithms on Strehl ratio and visual discrimination capacity after LASIK. In their study, the target Q in F-CAT algorithms was set -0.5 and optical and visual deterioration were greater after standard ablation. Except -0.20, other values of target Q was also reported. Koller et al. [12] considered that Q-factor customized ablation aiming for a Q-target of -0.40 was as effective as wavefront-guided ablation in correcting myopic astigmatism. In our study, target Q value was set according to the mean of Q_1_ and Q_2_, which were calculated by eccentricity of two main meridians within 30 degrees, fully considered each individual’s corneal asphericity before surgery. Since we did not carry out controlled trials with conventional ablation in this study, whether the F-CAT algorithm was effective in reducing the Q value shift after the surgery had not been completely verified. In addition, the corneal biomechanical response and the healing response might be the important factors [13], especially in high myopic astigmatism.

When correcting high myopia, how did the procedure impact the corneal astigmatism? We found Q value ablation could reduce corneal astigmatism with statistically significant differences between preoperative and postoperative values. The aspheric LASIK procedure led to more operative astigmatism compared to aspheric LASEK. In the LASIK procedure, we had to make a corneal flap to expose the stromal bed. The stroma flap procedure was probably the reason that additional astigmatism occurred. Therefore, the effects of correcting astigmatism were not satisfactory.

Furthermore, refractive surgery inevitably changed the regularity of the cornea. We could find that all the indices which reflected corneal regularity increased dramatically. These indices included ISV, IVA and IHA. They all reflected the regularity of the cornea following surgery after 3 years. There were no statistically significant differences between aspheric LASIK and aspheric LASEK. Either type of surgery increased the corneal irregularity.

In the present study, we analyzed high order aberrations of the anterior corneal surface, not including the posterior corneal surface and lens aberrations or other internal aberrations. We think that the changes of the anterior corneal surface could more accurately reflect the effects of LASIK and LASEK refractive procedures. Previous study has reported that changes of the anterior corneal surface could reflect on the optical quality of the visual system [14]. In high order aberrations, we focused on the observations of spherical aberration and coma, the two main HOAs that mostly impact visual quality, not including the total HOA RMS(Root Mean Square). As is commonly accepted, the same total HOAs may have different individual Zernike terms with different visual performances [15].

As shown in this study, both aspheric LASIK and aspheric LASEK significantly increased spherical aberration and coma at a 6 mm pupil parameter mode compared with preoperative values. The LASIK group exhibited significantly smaller changes in the spherical aberration (Z_4_^0^) than the LASEK group. We think that was due to a greater induction of HOAs after a LASEK epithelial flap compared to a LASIK stromal flap because of differing cytokine regulation. It is proposed that these differences are primarily due to the different methods of operation. A LASEK epithelial flap and a LASIK stromal flap have different biomechanical changes and postoperative healing responses, which may cause these differences. An epithelial flap has greater postoperative wound remodeling, which may alter HOAs in a different manner. Buzzonetti et al. [16] reported similar results. They compared corneal aberrations after LASIK and LASEK procedures for myopia. Coma-like and spherical-like aberrations increased in both groups, but spherical-like aberrations were greater after LASEK than LASIK over a 3 mm pupil.

According to correlation and regression analysis results, the main impact factors of spherical aberration after surgery were optic zone size, changes in Q value, group and changed refraction . As we all know, spherical aberration after surgery was closely related to the optic zone size of the excimer laser ablation. Setting a larger optical zone size produced less spherical aberration. Furthermore, the changes in Q value had a positive correlation with spherical aberration after surgeries. The difference between the two groups regarding the influence of Z_4_^0^ was stated above. The R^2^ of this model was 0.644 and therefore we think this model had enough strength to explain the relation between spherical aberration and its impact factors.

Another important aberration that influenced visual quality after surgery was coma. It was widely believed that eccentric ablation was the main reason for coma aberration formation [17]. Our results showed the coma of the anterior corneal surface increased following surgery at year 3 in the two groups, with no difference between them. The main impact factors of coma were gender, optic zone size and MRSE prior to surgery. Female subjects had more coma of the anterior corneal surface than males. It was worth noting that there were more females than males in our study. The unbalanced sex ratio might cause a bias in the statistical test. In addition, according to the linear model, the greater the spherical equivalent before surgery and the smaller the optic zone size of excimer laser ablation were, the more risk for coma after surgery.

## Conclusions

In the current study, our results indicated that Q value guided surgery had ideal safety, efficacy and predictability after 3 years. Both Q value guided LASIK and LASEK procedures caused increases in asphericity (Q value), corneal regularity indices, corneal astigmatism, spherical aberration and coma for high myopia astigmatism treatment. Postoperative anterior corneal spherical aberration was greater following LASEK than LASIK and the anterior corneal astigmatism was greater following LASIK than LASEK. As the same excimer laser treatment (F-CAT algorithm) was applied, it was presumed that the differences were related to the methods of operation and healing responses. The primary impact factors of spherical aberration included optic zone size, changes of Q value, operation methods (LASIK or LASEK) and corrected refraction.

## Competing interests

The authors declare that they have no competing interests.

## Authors’ contributions

HH participated in the design of the study, performed the statistical analysis and drafted the manuscript. JZ conceived of the study and participated in its design and coordination and helped to revise the manuscript. JY, HB, SC, BX carried out the data collection. All authors read and approved the final manuscript.

## Pre-publication history

The pre-publication history for this paper can be accessed here:

http://www.biomedcentral.com/1471-2415/12/15/prepub
